# Management of Multiple Sclerosis in the Breastfeeding Mother

**DOI:** 10.1155/2016/6527458

**Published:** 2016-02-04

**Authors:** Saneea Almas, Jesse Vance, Teresa Baker, Thomas Hale

**Affiliations:** ^1^Department of Pediatrics, School of Medicine, Texas Tech University Health Sciences Center, 1400 Wallace Boulevard, Amarillo, TX 79106, USA; ^2^Infant Risk Center, Texas Tech University Health Sciences Center, 1400 Wallace Boulevard, Amarillo, TX 79106, USA; ^3^Department of Obstetrics & Gynecology, School of Medicine, Texas Tech University Health Sciences Center, 1400 Coulter Street, Amarillo, TX 79106, USA

## Abstract

Multiple Sclerosis (MS) is an autoimmune neurological disease characterized by inflammation of the brain and spinal cord. Relapsing-Remitting MS is characterized by acute attacks followed by remission. Treatment is aimed at halting these attacks; therapy may last for months to years. Because MS disproportionately affects females and commonly begins during the childbearing years, clinicians treat pregnant or nursing MS patients. The intent of this review is to perform an in-depth analysis into the safety of drugs used in breastfeeding women with MS. This paper is composed of several drugs used in the treatment of MS and current research regarding their safety in breastfeeding including immunomodulators, immunosuppressants, monoclonal antibodies, corticosteroids, and drugs used for symptomatic treatment. Typically, some medications are large polar molecules which often do not pass into the milk in clinically relevant amounts. For this reason, interferon beta is likely safe for the infant when given to a breastfeeding mother. However, other drugs with particularly dangerous side effects may not be recommended. While treatment options are available and some data from clinical studies does exist, there continues to be a need for investigation and ongoing review of the medications used in breastfeeding mothers.

## 1. Introduction

Multiple Sclerosis (MS) is a common neurological disease in young adults, affecting approximately 400,000 individuals in the United States and over 2 million people worldwide [1]. MS is an autoimmune disease characterized by both diffuse and localized inflammation, demyelination of neurons, and nonspecific brain and spinal cord damage [2]. The course of the disease is variable and patients commonly experience a period of clearly defined attacks followed by periods of complete or partial recovery. This type of MS is classified as Relapsing-Remitting MS (RRMS), and it accounts for nearly 85% of all cases [3, 4]. During attacks, patients may experience deficits in any number of systems (motor, sensory, optic, sphincteric, etc.) [5]. Treatment of MS is aimed at halting attacks when they occur. Treatments usually last for years. Nonspecific immunosuppressive agents are the mainstay of therapy [6]. The future of MS research will be aimed at repairing and reversing damage to the myelin sheath; however, the understanding of disease etiology is still limited [7].

As with most autoimmune diseases, MS disproportionately affects females with a threefold increased risk as compared to males. The common age of onset is during the third and fourth decades of life, coincidentally a woman's childbearing years [2, 8]. Due to medical advancements in the past two decades, clinicians have become more supportive of young adults with MS who choose to start a family. Because of the development of disease modifying drugs (DMDs), healthcare professionals have the ability to reduce the accumulation of CNS damage and resulting disability by extending the time between relapses. Women with MS have become more confident in their ability to safely and successfully become pregnant and have a healthy child. The therapeutic management of MS in the pregnant woman has been adequately covered in recent years [9, 10]. However, an in-depth investigation into the safety of DMDs in breastfeeding women and their infants is limited. Given that approximately 72% of women in the USA choose to breastfeed and up to 30% of women with MS may relapse within the first 3 months postpartum, the safety of medications used to treat MS while breastfeeding is of paramount concern to mothers and their infants [10, 11].

## 2. Transfer of Medications into Breast Milk

While the exact nature of the transfer of DMDs into breast milk is still largely unknown, we do have a reasonable system for estimating drug transfer in some cases [11, 12]. Newer agents often lack extensive research, but the relative risk to the infant of most medications can be estimated to some degree. While the benefits of breastfeeding an infant are enormous, the risk of incidental drug exposure to a certain drug may outweigh the benefits of breastfeeding. This risk assessment is the subject of this review. Finally, the absolute cessation of breastfeeding should only be recommended with drugs that have extremely hazardous side effects [13].

The transfer of a drug into breast milk is dependent upon many factors. These include molecular weight, protein binding, pKa, lipid solubility, volume of distribution, and the presence of any active transport mechanisms [14]. The drug's molecular weight is perhaps the most important determinate of its transfer into breast milk [15]. In general, large polar molecules often do not pass into milk in clinically relevant amounts. A drug's protein binding is also relevant because drugs that are highly protein bound are generally unable to pass into milk [16]. Drugs with high volumes of distribution (*V*_*d*_) are often concentrated in body tissues outside the plasma and produce limited plasma levels; therefore, they are often considered safer for breastfeeding mothers. Oral ingestion of a drug in milk is followed by digestion in the gut and first-pass metabolism through the liver. Drugs with high first-pass effect are often sequestered in the liver and fail to produce high plasma levels. As the infant ages, drug metabolism and first-pass effect increase [17]. Local effects on the gastrointestinal tract of the infant should be considered when relevant. Other risk factors that should be considered are age (<4 months), prematurity or complications during delivery, gastrointestinal anomalies, and the presence of metabolic problems in the infant. Younger infants are characteristically at higher risk than older (>9 months) infants.

The Relative Infant Dose (RID) is another frequently used tool to evaluate clinical risk to the infant. The RID is calculated using the mother's dose, the concentration of drug in milk, and the volume of breast milk consumed by the infant. In infants that exclusively breastfeed, the RID is an estimate of the amount of mother's daily medication that the infant receives via milk. For example, if the mother takes 100 mg of a drug with RID of 1%, the infant will be exposed to about 1 mg. Most experts in breastfeeding medicine consider RID of 10% to be a reasonable cutoff between safe and nonsafe, assuming that the drug in question does not have extraordinary toxicity associated with it [18]. However, each case should be carefully considered before making a clinical recommendation.

## 3. Multiple Sclerosis and Breastfeeding

In 2002, a study suggested that mothers with MS who wished to breastfeed their newborns could safely do so; however, the authors advised against the use of immunomodulating drugs while nursing and suggested postponing breastfeeding for three months after the last dose of any disease modifying agent [19]. Several studies have attempted to assess, with conflicting results, whether or not breastfeeding itself affects the disease or the rate of relapse in MS patients. A recent meta-analysis concluded that women who breastfeed are half as likely to experience a relapse compared to women who do not breastfeed. While this study cites limitations of heterogeneity and adequate breastfeeding recall, it also mentions that women with severe disease might be less likely to breastfeed due to disease limitations [20]. Further research is needed in order to adequately determine the existence of any association between breastfeeding and MS relapse.

Many studies have failed to consider the differences between exclusive breastfeeding and breastfeeding supplemented with formula feeds. It is well known that exclusive breastfeeding results in different hormonal changes in women compared to those who only partially breastfeed and use formula supplements. One study reported a beneficial effect of exclusive breastfeeding on Multiple Sclerosis. This study also suggests that lactational amenorrhea could contribute to a reported decrease in relapse rate [21].

Another study suggests that patients who are not on immunomodulatory therapy are more likely to experience MS exacerbation within three months of giving birth. This study concluded that there was no significant effect of breastfeeding on the patient's risk of experiencing a relapse and that the relapse rate returned to prepregnancy levels at three months postpartum regardless [22]. A multicenter study following 423 pregnancies in women with MS over the course of six years concluded that postpartum relapses were more likely found in women with higher disease activity before pregnancy. The study recommended early resumption of Disease Modifying Drugs in patients with severe disease [23].

Considering current evidence, the choice of becoming pregnant and breastfeeding are both possible and safe options for women with MS. However, for women actively experiencing severe disease symptoms, treatment should probably be restarted. While the safety of disease modifying drugs in pregnancy is somewhat understood, their safety in the infant following breastfeeding has not been well established.

## 4. Medications Used for Multiple Sclerosis: Description, Adverse Effects, and Safety during Breastfeeding

### 4.1. Immunomodulators

#### 4.1.1. Interferon Beta

Interferon beta including interferons beta 1a and beta 1b is first-line therapy for MS. Various dosage formulations are available. While the mechanism whereby interferons work remains to be fully understood, their anti-inflammatory properties are well documented. Several studies have suggested significant modulatory effects on the immune system. Interferon beta is known to alter cell surface receptor interactions, possibly contributing to the immunosuppressive effects on Relapsing-Remitting MS [24]. Still, the exact mechanism in the treatment of MS is still unknown. Interferon beta 1b is also used to treat secondary progressive MS. Treatment with interferon beta produces side effects of flu-like symptoms, injection site reactions, headache, fatigue, and elevated ALT levels [25].

Interferon beta 1a's therapeutic effects are noted within 12 hours after the initial dose. This effect lasts 4 days. Its half-life is approximately 19 hours, with a time-to-peak serum concentration of 5 hours. The half-life of interferon beta 1b ranges from 8 minutes to 4.3 hours. It requires 1–8 hours to reach peak serum concentrations. Interferon beta 1a is not as well studied as interferon beta 1b [26].

Small amounts are excreted in breast milk. In a recent study of the transfer of interferon beta 1a in six mothers receiving 30 *μ*g per week, average milk concentrations were 46.7, 97.4, 66.4, 77.5, 103.1, 108.3, 124, and 87.9 pg/mL at 0, 1, 4, 8, 12, 24, 48, and 72 hours, respectively, after dosing. The estimated Relative Infant Dose would be 0.006% of the maternal dose. Interferons have a large molecular weight, bind to T cells, are distributed out of the plasma compartment, and are relatively nontoxic. No adverse events have been reported in the nursing infant. Interferon beta is currently classified as probably compatible with limited data for breastfeeding mothers [27, 28].

#### 4.1.2. Fingolimod

Fingolimod is rapidly metabolized to fingolimod phosphate which inhibits sphingosine-1-phosphate receptors. This may reduce lymphocyte transfer into the central nervous system. Fingolimod is an immune modulator and prodrug that binds to the surface of lymphocytes and redirects them from the blood and graft sites to the lymph nodes, thus reducing the immune response in patients with Multiple Sclerosis. It reportedly assists in the repair of brain glial and precursor cells following injury. It slows the progression of disability and reduces the frequency and severity of symptoms in patients with MS. Side effects of therapy include headache, cough, macular edema, weakness, dizziness, hypotension, bradycardia, diarrhea, flu-like symptoms, increased hepatic enzyme levels (ALT and AST), and back pain. Hourly monitoring is required after the first dose of fingolimod for bradycardia and hypotension detection [29].

Fingolimod has *V*_*d*_ of 1200 L and is also highly protein bound in the plasma compartment. Oral bioavailability is high at 93%. Elimination half-life is 6 to 9 days, and peak concentration levels are reached 12–16 hours after dosing [30]. It is unknown if fingolimod passes into human breastmilk, but it is known to be excreted into rat milk. Due to its high volume of distribution and high protein binding, levels in human milk will probably be low. While it is less likely to be found in breastmilk in significant amounts, because of its significant risk of cardiovascular side effects, it is categorized as hazardous at this time.

#### 4.1.3. Teriflunomide

Teriflunomide is a pyrimidine synthesis inhibitor and may exert its effects against MS by reducing the number of activated lymphocytes in the central nervous system. Adverse effects of therapy include headache, hypophosphatemia, neutropenia, lymphocytopenia, increased hepatic enzyme levels (ALT), and influenza infection [31].

Teriflunomide has a volume of distribution of 11 L/kg, and it is extensively protein bound at 99%. Its half-life is 18-19 days with peak serum concentration at 1–4 days. No human studies have been conducted regarding this drug's ability to transfer into human milk. Animal studies have identified teriflunomide in rat milk after a single dose but rodent studies are not at all indicative of human transfer.

Given this drug's toxicity in adults, its likely presence in milk, and its long half-life, teriflunomide should be used with great caution in breastfeeding mothers. It is categorized as hazardous with no data.

### 4.2. Immunosuppressants

#### 4.2.1. Glatiramer Acetate

Glatiramer acetate is a synthetic polymer of amino acids L-tyrosine, L-glutamate, L-alanine, and L-lysine. Glatiramer acetate is similar in composition to the myelin sheath of nerves. It is believed to exert its effects by binding to the major histocompatibility class II molecule. Side effects in patients receiving therapy include chest pain, diaphoresis, skin rash, injection site residual mass, dyspnea, and flu-like symptoms.

Small amounts of the drug are believed to enter the lymphatic circulation. Its molecular weight ranges from 4,700 to 13,000 Daltons. No data are available on its transfer into human milk, but, due to its large molecular weight, transfer is very unlikely [32]. It is not known to cross the blood-brain barrier, and it is unlikely to cross into the breast milk. After oral ingestion, it is depolymerized into individual amino acids so oral bioavailability in the breastfed infant would be nil [33]. It is currently classified as probably compatible with no data.

#### 4.2.2. Mitoxantrone

Mitoxantrone intercalates itself into DNA and inhibits DNA topoisomerase II, resulting in decreased DNA synthesis and replication. It also alters RNA synthesis. Side effects of mitoxantrone therapy include edema, arrhythmia, alopecia, amenorrhea, sepsis, and gastrointestinal disturbances, headache, fever, fatigue, cardiotoxicity, mucositis, anorexia, changes in liver and renal function, leukopenia, neutropenia, thrombocytopenia, pruritus, phlebitis, and increased frequency of infection [34].

Mitoxantrone has poor oral absorption with a large volume of distribution of 14 L/kg. Mitoxantrone is distributed into peripheral tissues and binds to proteins. The mean alpha half-life of mitoxantrone is six to twelve minutes, the mean beta half-life is 1.1 to 3.1 hours, and the mean gamma (terminal or elimination) half-life is 23 to 215 hours (median = 75 hours). Distribution to tissues is extensive: steady state volume of distribution exceeds 1,000 L/m^2^. In a study of a patient who received 3 treatments of mitoxantrone (6 mg/m^2^) on days 1 to 5, mitoxantrone levels in milk measured 120 ng/mL just after treatment and dropped to a stable level of 18 ng/mL for the next 28 days [35]. Assuming that a mother was breastfeeding, these levels would provide about 18 *μ*g/L of milk consumed after the first few days following exposure to the drug. In addition, it would be sequestered for long periods in the infant as well. The RID is estimated to be 2–12% of the maternal dose. Due to its significant side effect profile, it is currently classified as hazardous with limited data and should probably not be used by breastfeeding mothers [36].

#### 4.2.3. Dimethyl Fumarate

Dimethyl fumarate exerts its effects by modifying the cellular response to oxidative stress. The exact mechanism of action is unknown, although it is believed to produce anti-inflammatory properties [37]. Adverse reactions associated with dimethyl fumarate include flushing, abdominal pain, infection, diarrhea, abdominal pain, changes in liver enzymes, nausea, proteinuria, rash, lymphopenia, and contact dermatitis [38].

Dimethyl fumarate has a volume of distribution of 53 to 73 L/kg and an active metabolite with a low molecular weight (129 D). It is metabolized very quickly with a half-life of approximately 1 hour. Time required to reach peak serum concentration is 2 hours [39]. Its high volume of distribution and rapid metabolism would likely preclude significant transfer into human milk. However it is categorized as possibly hazardous with no data due to its significant side effect profile. The active drug component (monomethyl fumarate) has a low molecular weight of only 129 Daltons and low protein binding (27–45%). This product will probably enter milk significantly. Until more is known about its transfer into milk, caution is recommended for its use in breastfeeding mothers.

#### 4.2.4. Cladribine

Cladribine, a prodrug that is phosphorylated intracellularly, is an immunosuppressive agent that induces irreparable DNA damage within cells and induces apoptosis of lymphocytes. Side effects of therapy include fever, fatigue, headache, rash, appetite suppression, neutropenia, anemia, thrombocytopenia, abnormal breath sounds, and infections. Local injection site reactions may have also been reported [40].

Cladribine has a volume of distribution of <9 L/kg indicating extensive sequestration in body tissues and is 20% bound to protein with a relatively low molecular weight of 285 D. Its half-life is about 5 hours, with some estimates as high as 19 hours. It is unknown if cladribine enters breast milk, but it enters cerebrospinal fluid significantly (25% of plasma levels), suggesting it might easily transfer into human milk as well. It is rated as hazardous in breastfeeding mothers, and it is recommended to withhold breastfeeding for 48 hours after a dose of cladribine and longer in the event of maternal renal dysfunction.

#### 4.2.5. Azathioprine

Azathioprine is a derivative of mercaptopurine. It exerts its effects via its metabolites which halt DNA replication and purine synthesis. Side effects of therapy with azathioprine include malaise, nausea, vomiting, leukopenia, and increased susceptibility to infection [41, 42]. Azathioprine is 30% bound to plasma proteins. Its half-life is approximately 2 hours.

In two mothers receiving 75 mg azathioprine, the concentration of 6-MP in milk varied from 3.5–4.5 *μ*g/L to 18 *μ*g/L [43]. The authors conclude that these levels would be too low to produce clinical effects in a breastfed infant. In another study of two infants who were breastfed by mothers receiving 75–100 mg/day azathioprine, both infants had normal blood counts, no increase in infections, and above-average growth rate [44].

Four mothers who were receiving 1.2–2.1 mg/kg/day of azathioprine throughout pregnancy and continued postpartum were studied, and neither 6-TGN nor 6-MMPN could be detected in the exposed infants [45]. Four case reports were performed with mothers taking between 50 and 100 mg/day of azathioprine and reported no adverse events in any of the infants [46].

Ten women at steady state on 75 to 150 mg/day azathioprine provided milk samples on days 3-4, days 7–10, and day 28 after delivery; 6-MP and 6-TGN were undetectable in the infants' blood [47]. Another study of three mothers taking azathioprine while breastfeeding (doses of 100–175 mg) reported normal blood cell counts in all three infants [48]. In a group of 8 lactating women who received azathioprine (75–200 mg/day), levels in milk ranged from 2 to 50 *μ*g/L. The authors estimate the infant's dose to be <0.008 mg/kg/day [49].

In a 31-year-old mother with Crohn's disease being treated with 100 mg/day azathioprine, peripheral blood levels of 6-MP and 6-TGN in the infant were undetectable at day 8 or after 3 months of therapy [50]. In a recent study of the long-term follow-up (median 3.3 years) of fetal and breastfeeding exposure to azathioprine (*n* = 11 infants), there were no differences in rates of infectious disease in azathioprine-treated groups compared to nontreated controls [51].

In summary, azathioprine enters breast milk at RID of 0.07–0.3% and is categorized as probably compatible with limited data. Although no adverse effects have been reported, breastfed infants of mothers taking azathioprine should be monitored for signs of leukopenia, pancreatitis, and immunosuppression.

#### 4.2.6. Cyclophosphamide

Cyclophosphamide is an alkylating agent that prevents cell division by cross-linking DNA strands and decreasing DNA synthesis. Cyclophosphamide is a potent immunosuppressant. It is well absorbed orally and has a volume of distribution from 30 to 50 L/kg. Half-life is 3–12 hours and time-to-peak serum concentration is 1–3 hours after a dose. Side effects of cyclophosphamide therapy are significant and include alopecia, amenorrhea, gonadal suppression, abdominal pain, hemorrhagic cystitis, anemia, and dose dependent leukopenia [52].

Cyclophosphamide is known to enter breast milk. In one case of a mother who received 800 mg/week of cyclophosphamide, the infant was significantly neutropenic following 6 weeks of exposure via breastmilk [53]. In another mother who was receiving 10 mg/kg intravenously daily for seven days for a total of 3.5 gm, major leukopenia was also reported in her breastfed infant. Thus far, no reports have provided quantitative actual estimates of cyclophosphamide in milk [53, 54]. Regardless, cyclophosphamide is categorized as hazardous, with no data, and it should be avoided in breastfeeding mothers. Mothers should withhold breastfeeding for a period of at least 72 hours after the last dose.

#### 4.2.7. Methotrexate

Methotrexate is used off the label for the treatment of MS. It inhibits dihydrofolate reductase and prevents DNA synthesis. Adverse effects associated with methotrexate therapy include chest pain, headache, dizziness, alopecia, skin photosensitivity, cerebral thrombosis, azotemia, thrombocytopenia, aplastic anemia, and increased liver enzymes [55].

Up to 3–6 weeks may be required to produce a significant therapeutic effect. Methotrexate has a volume of distribution from 0.18 to 0.8 L/kg. It is 50% bound to protein. Half-life is 3–10 hours for lower doses and 8–15 hours for higher doses. In children, the half-life is 1–6 hours. Time-to-peak serum concentration ranges from 1 to 2 hours after administration [56].

Methotrexate is secreted in breast milk in small amounts [57]. In 1972, a case report was published concerning a woman receiving 22.5 mg/day oral dose of methotrexate. Two hours after dose, the methotrexate concentration in breastmilk was 2.6 *μ*g/L of milk. The cumulative excretion of methotrexate in the first 12 hours after oral administration was only 0.32 *μ*g in milk [58]. In 2014, a second case report was published about a woman who was taking 25 mg/week of methotrexate for rheumatoid arthritis. This woman reinitiated her therapy 151 days postpartum because her disease worsened. The maternal serum concentration was 0.92 *μ*M 1 hour after her dose was given; breastmilk samples taken at 2, 12, and 24 hours after her dose were 0.05 *μ*M (detectable but below the level of quantification). The authors estimated the average infant dose to be 3.4 *μ*g/kg/day (22.7 *μ*g/L). This infant continued to breastfeed at 5 months of age when methotrexate was reinitiated in the mother and for another 9 months. No adverse events were reported in the infant [59].

Methotrexate does enter into breast milk with RID of 0.1 to 0.9%. It is categorized as possibly hazardous with limited data. If the mother breastfeeds more than 24 hours after the last dose of methotrexate, the infant should be monitored for vomiting, diarrhea, and blood in the vomit, stool, or urine [60]. In extreme cases where the infant cannot be withdrawn from the breast, the infant could be supplemented with folic acid or L-methylfolate.

### 4.3. Monoclonal Antibodies

#### 4.3.1. Natalizumab

Natalizumab is a monoclonal IgG4k antibody that binds to the *α*4-subunit of *α*4*β*1 and *α*4*β*7 integrin molecules. Integrins are responsible for adhesion and subsequent migration of cells from the bloodstream to the site of inflammation in tissues. Natalizumab prevents the transmigration of leukocytes to inflamed tissues by binding to the alpha-4 subunit on the surface of leukocytes. In Multiple Sclerosis, it is believed to block T lymphocyte migration into the CNS resulting in a decreased rate of relapse. Natalizumab is primarily indicated for patients with moderate-to-severe relapsing forms of MS. Side effects of natalizumab therapy include headache, fatigue, depression, rash, gastrointestinal disturbances, urinary tract infections, flu-like symptoms, and progressive multifocal leukoencephalopathy [61].

The volume of distribution of natalizumab is 3.8–7.6 L/kg and half-life is 7–15 days. Due to the prolonged time required to achieve steady state (28 weeks), actual concentration in breast milk at steady state is still uncertain at this time. One recent study demonstrated rising levels of natalizumab in breastmilk in one patient with an estimated RID of 2–5%. However, this study only followed the infants for 50 days and natalizumab requires up to 28 weeks to achieve steady state [62]. Therefore, the authors suggest that further research is needed to evaluate levels at steady state and risks to the breastfed infant. Although no adverse effects were reported in the infant, it is categorized as probably compatible but with limited data.

#### 4.3.2. Alemtuzumab

Alemtuzumab is a recombinant DNA-derived humanized monoclonal antibody that depletes T and B lymphocytes by targeting CD52 glycoproteins on T and B cells, lymphocytes, monocytes, macrophages, and natural killer cells. It is indicated for patients with relapsing MS who have had inadequate response to other products. Adverse effects of therapy with alemtuzumab are significant and include autoimmune diseases, fever, headache, insomnia, paresthesia, skin rash, thyroid dysfunction, and urinary tract infections. The most significant side effect is lymphopenia. Infusion related reactions and infections are frequently reported [63, 64].

The volume of distribution is different for the different formulations of alemtuzumab. Intravenous alemtuzumab has *V*_*d*_ of 0.18 L, while the oral formulation has *V*_*d*_ of 4.1 L. The half-life of the intravenous formulation is 11 hours and oral formulation is approximately 2 weeks. Because it is a monoclonal antibody and because it has a large molecular weight (150 kD), alemtuzumab is unlikely to significantly enter breast milk in clinically relevant amounts. Despite the fact that maternal IgG is known to transfer into breast milk at very low levels, because of its significant side effect profile, alemtuzumab is categorized as possibly hazardous.

#### 4.3.3. Rituximab

Rituximab is a chimeric (human/mouse) monoclonal antibody that targets CD20 receptors on B lymphocytes. Adverse reactions seen with rituximab therapy include hypertension, fever, fatigue, headache, insomnia, rash, cytopenia, and increased hepatic ALT levels. Neuropathy and muscle spasms have also been reported. Infusion related reactions may also be seen with rituximab therapy [65].

Rituximab has a volume of distribution of 3.1 to 4.1 L/kg. Half-life ranges from 18 to 32 days. It remains in the plasma for 3–6 months after the last dose. Although it is unlikely to enter breastmilk due to its large molecular weight, the long half-life and significant side effect profile have led it to be categorized as possibly hazardous with no data.

#### 4.3.4. Daclizumab

Daclizumab is a humanized monoclonal antibody that targets IL-2 and CD25. It has a relatively long half-life, ranging from 21 to 25 days in healthy individuals and is highly bioavailable after subcutaneous injection. Hypersensitivity is a known side effect and patients treated with daclizumab often experience cutaneous adverse effects. Other side effects include arterial hypertension, dyspnea, and edema [66].

With a low volume of distribution of 2.5 L/kg, daclizumab probably remains in the plasma compartment for a significant amount of time [67]. It has a molecular weight of 144 kD and has somewhat limited potential to pass into breast milk [68]. Because it is unknown if daclizumab enters breast milk, this drug should be avoided pending further investigation.

### 4.4. Corticosteroid

#### 4.4.1. Methylprednisolone

Methylprednisolone is a typical corticosteroid. Corticosteroids exert their effects by regulating gene expression and modify carbohydrate, protein, and lipid metabolism. The therapeutic effects of methylprednisolone in MS are believed to be due to its anti-inflammatory properties [69, 70]. Although rare, corticosteroids may produce significant side effects including cardiovascular (arrhythmia, bradycardia, and cardiomegaly), neurological (delirium, depression, and hallucinations), dermatological (acne, alopecia), endocrine (adrenal suppression, diabetes mellitus), and gastrointestinal (abdominal distention, pancreatitis, and peptic ulcer disease) effects when used for prolonged periods.

Oral methylprednisolone reaches its peak effect at 1-2 hours after administration, while IM administration can take up to 4–8 days [71, 72]. Methylprednisolone's volume of distribution is 0.7 to 1.5 L/kg. The half-life of methylprednisolone is about 3 hours, and it is reduced in obese individuals [73].

In a recent case report of a 36-year-old woman who received methylprednisolone 1000 mg IV daily × 3 days for treatment of relapsing MS, the authors found that the infant would ingest only 0.164 to 0.207 mg/kg/day of methylprednisolone. Levels in milk were low and dissipated by 8–12 hours. Oral methylprednisolone is secreted into breast milk with RID of only 0.4 to 3%. Based on this data, it would seem reasonable to continue breastfeeding while receiving a short course of high-dose methylprednisolone. If the mother wishes to further limit infant exposure, she should interrupt breastfeeding for 8–12 hours after high intravenous doses [74]. Methylprednisolone use in breastfeeding women is probably compatible although with limited data.

### 4.5. Symptomatic Treatments

#### 4.5.1. Dalfampridine

Dalfampridine is a potassium channel blocker that is approved to improve gait in patients with disability caused by MS. Dose-related seizures have been reported [13]. While it is a broad spectrum potassium channel blocker, it apparently does not increase QTc interval. Other adverse reactions to therapy include urinary tract infections, anaphylaxis, insomnia, dizziness, headache, nausea, and vomiting.

Dalfampridine has a limited volume of distribution of 2.6 L/kg. It is not bound to proteins and its absorption is rapid and complete. Its half-life is 5-6 hours but it is prolonged in patients with renal impairment. The drug requires 3-4 hours to reach peak plasma concentrations. Because dalfampridine has a small molecular weight of only 94 Daltons, it is probably a highly risky product to use in breastfeeding mothers [75]. The kinetics of this product are ideal for entering milk at high levels. Caution is recommended. This product is well known to be highly toxic in some animal species (birds). This drug should be avoided in breastfeeding mothers pending further investigation [76].

#### 4.5.2. Baclofen

Baclofen inhibits spinal cord reflexes and reduces spasticity. While its exact mechanism of action is unknown, it apparently acts as an agonist at presynaptic GABA-B receptors at the level of the spinal cord. It is approved for the treatment of spasticity in MS patients. Side effects of therapy include drowsiness, excitement, dry mouth, urinary retention, tremor, confusion, headache, hypotension, rigidity, and wide pupils. Muscle rigidity, exaggerated spasticity, multiple system organ failure, and death have been reported.

Baclofen can be administered intrathecally or intravenously. Intrathecal onset of action is quick, about 30 minutes to 1 hour, while IV onset requires 6–8 hours. Peak effect is attained at 4 hours with intrathecal administration, 24–48 hours with IV infusion, and 2 hours with oral administration. Baclofen is 30% protein bound, with a molecular weight of only 214 Daltons and a half-life of 2–4 hours [58].

Small amounts of baclofen are known to be secreted into milk. In one mother given a 20 mg oral dose, total consumption by infant (via milk) over a 26-hour period was estimated to be 22 *μ*g, about 0.1% of the maternal dose. Milk levels ranged from 0.6 *μ*mol/L to 0.052 *μ*mol/L at 26 hours. The maternal plasma and milk half-lives were 3.9 hours and 5.6 hours, respectively. It is unlikely that baclofen administered intrathecally would transfer into milk in clinically relevant quantities [77]. The RID is estimated at 6.9% of the maternal dose. No adverse effects were seen in the infant of the breastfeeding mother, and it is categorized as probably compatible with limited data.

## 5. Conclusion

Women undergoing treatment for MS will often be faced with the decision as to which drugs are safe while breastfeeding.  Table 1 summarizes the list of medications used in MS patients and their descriptions and RID and pertinent clinical considerations. Healthcare professionals should encourage women with MS to breastfeed just as they would encourage healthy mothers after each individual patient's health status and medications are reviewed. Because it is likely that a woman will suffer a relapse in the first few months postpartum, the decision to continue or withhold breastfeeding should be made by the physician as well as the patient after weighing the risks and benefits. With an appropriate therapeutic approach, breastfeeding does not have to be stopped in all occasions. Each clinician should help the patient make the correct decision using accurate information provided here and in other sources.

## Figures and Tables

**Table 1:** 

Drug	Description	RID (%)	Clinical considerations
Interferons b1a and b1b^*∗*^	Immunomodulator and antineoplastic. Differs slightly from naturally occurring proteins. Anti-inflammatory properties	0.006	Limited data, probably *compatible*. Large MW. Relatively nontoxic

Glatiramer acetate^*∗*^	Immunosuppressant. Synthetic polypeptide of the amino acids L-alanine, L-glutamic acid, L-lysine, and L-tyrosine	—	No data, probably *compatible*. Large MW of 4,700 to 13,000 Daltons likely prohibits entry into breast milk. Toxicity unlikely

Mitoxantrone^*∗*^	Immunosuppressant and antineoplastic. DNA intercalating agent inhibits topoisomerase II	2–12	Limited data, *hazardous*. Large *V*_*d*_ and half-life may lead to sequestering of drug in infant even with limited exposure via breast milk

Natalizumab^*∗*^	Recombinant monoclonal antibody	5	Limited data, probably *compatible*. Due to long time required to achieve steady state, actual concentration in breast milk is unknown

Fingolimod^*∗*^	Sphingosine-1-phosphate (SIP) modulator	—	No data, *hazardous*. High *V*_*d*_ and levels in breast milk are expected to be low. Hazardous product due to bradycardia and hypotension as significant adverse effects. Hourly monitoring required

Dalfampridine^*∗*^	Potassium channel blocker improves skeletal muscle conduction—symptomatic treatment	—	Hazardous pending research. *V*_*d*_ 2.6 L/kg. 96% oral bioavailability. Unknown if enters breast milk. *Decide on case-by-case basis*

Baclofen^*∗*^	Inhibits spinal cord reflexes resulting in reduced spasticity—symptomatic treatment	6.9	Limited data, probably *compatible*. Enters breast milk in small amounts. No adverse effects seen in infant

Dimethyl fumarate^*∗*^	Ester derivative of fumaric acid, immunosuppressant	—	No data, possibly *hazardous*. Low MW of 129 Daltons makes it likely to enter breast milk. Monitor infant for vomiting and diarrhea

Alemtuzumab^*∗*^	Recombinant DNA-derived humanized monoclonal antibody targets CD52 glycoprotein	—	No data, possibly *hazardous*. MW of 150 kD. Unlikely to enter milk due to large size

Teriflunomide^*∗*^	Pyrimidine synthesis inhibitor Immunomodulator	—	No data, *hazardous*. Extensive half-life and GI absorption. Likely present in milk. Due to toxicity in adults, caution advised during breastfeeding

Cladribine	Immunosuppressive agent causes apoptosis of lymphocytes	—	No data, *hazardous*. Penetrates CSF with doses 25% of plasma. Potentially enters milk. Withhold breastfeeding for 48 hours or longer if maternal renal dysfunction exists

Methylprednisolone	Corticosteroid. Anti-inflammatory agent	0.4–3	Limited data, probably *compatible*. Enters breast milk in very low amounts

Azathioprine	Derivative of mercaptopurine; metabolites halt DNA replication and purine synthesis. Immunosuppressive agent	0.07–0.3	Limited data, probably *compatible*. No adverse effects seen in infants. Monitor infant for signs of leukopenia, pancreatitis, and immunosuppression

Rituximab	Chimeric (human/mouse) monoclonal antibody targets CD20	—	No data, possibly *hazardous*. Large MW, unlikely to enter milk. Significant side effect profile

Daclizumab	Humanized monoclonal antibody targets IL-2 and CD25	—	Not known if enters milk but probably minimal. *Hazardous* pending research. *Decide on case-by-case basis*

Cyclophosphamide	Antineoplastic agent suppresses DNA synthesis	—	No data, *hazardous*. Enters breast milk. Reports of leukopenia in infants; withhold breastfeeding for 72 hours

Methotrexate	Inhibits dihydrofolate reductase and prevents DNA synthesis	0.1–0.9	Limited data, possibly *hazardous*. Enters into breast milk. Contraindicated in breastfeeding due to potential for serious side effects. Monitor infant for vomiting, diarrhea, and blood in the vomit, stool, or urine

RID = Relative Infant Dose, MW = molecular weight. *V*_*d*_ = volume of distribution. MS = Multiple Sclerosis. IL = Interleukin. CD = Cluster of Differentiation. *∗* = FDA approved for MS Treatment.
